# Fatty Liver Index and Its Association with 10-Year Atherosclerotic Cardiovascular Disease Risk: Insights from a Population-Based Cross-Sectional Study in China

**DOI:** 10.3390/metabo13070850

**Published:** 2023-07-14

**Authors:** Jing Zhou, Jing Fan, Xiaoyun Zhang, Lili You, Diaozhu Lin, Chulin Huang, Feng Li, Kan Sun

**Affiliations:** Department of Endocrinology, Sun Yat-Sen Memorial Hospital, Sun Yat-Sen University, 107 Yanjiang West Road, Guangzhou 510120, China; zhoujing23@mail.sysu.edu.cn (J.Z.); fanj7@mail2.sysu.edu.cn (J.F.); zhangxy375@mail.sysu.edu.cn (X.Z.); youlli@mail.sysu.edu.cn (L.Y.); lindzh6@mail.sysu.edu.cn (D.L.); huangchl7@mail.sysu.edu.cn (C.H.); lifeng6@mail.sysu.edu.cn (F.L.)

**Keywords:** fatty liver index, non-alcoholic fatty liver disease, NAFLD, lipid metabolism, atherosclerotic cardiovascular disease, atherosclerosis

## Abstract

This cross-sectional study aimed to investigate the association between non-alcoholic fatty liver disease (NAFLD) and atherosclerotic cardiovascular disease (ASCVD), a global public health concern. A total of 9044 out of 10,104 adults from Guangzhou, China, were included in the analysis. We utilized the fatty liver index (FLI), a noninvasive indicator of NAFLD, and the pooled cohort equations (PCE) based on the 2013 ACC/AHA Guideline, the China-PAR model, and the Framingham Risk Score to assess the 10-year ASCVD risk. The results demonstrated a significant association between FLI and 10-year ASCVD risk (*p* < 0.001). Adjusted for age, individuals with high FLI (≥60) had an odds ratio of 3.91 (95% CI 2.52–6.08) compared to those with low FLI (<30). These findings persisted after adjusting for metabolic indicators. Notably, this association was consistently observed across all three risk prediction models: the PCE model, the China-PAR model, and the Framingham Risk Score. In conclusion, our study provides evidence supporting FLI as a reliable indicator of increased 10-year ASCVD risk in Chinese NAFLD patients. FLI serves as a valuable marker for early detection of ASCVD, highlighting its potential in clinical practice for risk assessment and prevention strategies.

## 1. Introduction

Non-alcoholic fatty liver disease (NAFLD), as the most widespread chronic liver disease, has been growing as a global public health concern. It encompasses a spectrum of conditions ranging from simple steatosis to metabolic-associated steatohepatitis, liver fibrosis/cirrhosis, and even hepatic carcinoma. NAFLD is estimated to occur among 20–30% of adults, and in 70–90% of individuals with obesity or diabetes [1,2]. Although previously considered to be of low incidence in Asia, the prevalence of NAFLD in the Asian population is now recognized as a growing concern, with rates in China estimated to range from 12 to 24%, having nearly doubled in the past 15 years due to the increasing incidence of obesity, T2DM, dyslipidemia, hypertension, and metabolic syndrome [3].

Recently, increasing evidence has linked NAFLD to an increased risk of atherosclerotic cardiovascular disease (ASCVD) and heart failure (HF) development [4,5]. Although some observational studies have shown inconsistent results, with NAFLD reported to have no relation to acute myocardial infarction or stroke after adjustment for cardiovascular disease (CVD) factors [6], there is a growing body of evidence linking NAFLD to increased ASCVD risk and severity.

To diagnose and prevent the progression of NAFLD and ASCVD, early identification of individuals with NAFLD is crucial. Liver biopsy is the most accurate method for evaluation and diagnosis, but it is invasive and uneconomical for large population samples. Ultrasound is a common noninvasive test but has limitations in detecting mild steatosis (affected by different operators) and accurately quantifying severity [7].

To address these limitations, aggregate scores based on routine clinical and biochemical data, such as the fatty liver index (FLI), have been proposed as noninvasive markers for early detection and management of NAFLD. FLI has been shown to be superior to other markers for the diagnosis of NAFLD during large epidemiological studies [8,9]. A study found that FLI outperforms other markers in terms of diagnostic and prognostic performance. FLI is based on easily obtainable clinical and biochemical data, including triglycerides (TGs), γ-glutamyl transferase (γ-GGT), body mass index (BMI), and waist circumference (WC). This makes FLI widely available in clinical practice. Furthermore, FLI has been validated by liver ultrasound scanning, magnetic resonance imaging, and liver biopsy in both the general population and in grade 3 obese persons. FLI also correlates with insulin resistance and can reliably predict the presence, not just the severity, of steatosis [8]. These findings indicate that FLI is an effective tool for diagnosing NAFLD.

Here we aimed to evaluate the association between FLI and the 10-year ASCVD risk in a community-based Chinese population. Traditional diagnostic methods for NAFLD have limitations in general detection. Thus, we analyzed data from a community-based Chinese population to investigate the role of FLI and its association with the prevalence of 10-year ASCVD risk. For the assessment of 10-year ASCVD risk, we utilized the 2013 American College of Cardiology/American Heart Association (ACC/AHA) Guideline to assess the cardiovascular risks, which utilizes the United States Pooled Cohort Equations (US-PCE) model [10]. The ACC/AHA model has been shown to be a superior tool in predicting primary prevention of ASCVD compared to other models, such as the SCORE or Framingham Risk Score, as demonstrated in the Copenhagen General Population Study [11]. The ACC/AHA model lowered the risk threshold for statin therapy in primary prevention to a 10-year absolute ASCVD risk of 7.5% [12], and has been found to be better calibrated in determining statin therapy decisions for populations like NAFLD [11]. By clarifying the association between FLI and the prevalence of 10-year ASCVD risk, we aim to contribute to the prevention and treatment of NAFLD.

In addition to utilizing the PCE model for the assessment of 10-year ASCVD risk, we also employed the traditional Framingham Risk Score and the 2016 China-PAR project assessment (Prediction for ASCVD Risk in China, 2016), using data from Chinese cohorts [12], which was specifically designed for the Chinese population. It is important to acknowledge that the common risk prediction models were primarily developed based on Caucasian populations. To evaluate the 10-year ASCVD risk prediction in our Chinese population, we incorporated the 2016 China-PAR model. This assessment has been shown to provide a more accurate prediction of ASCVD risk in the Chinese population. By incorporating these additional models, our aim was to gain further insights and assess the consistency of our findings with the PCE model.

## 2. Materials and Methods

### 2.1. Study Population and Design

**Figure 1:**
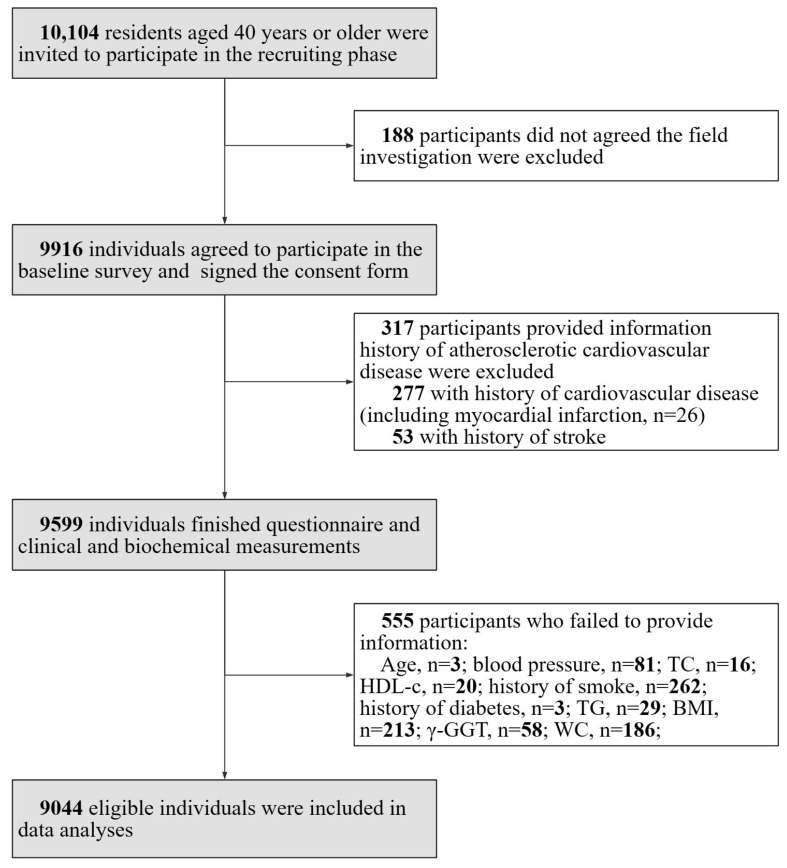
Flow chart of the study and the inclusion and exclusion criteria were shown.

The study was a cross-sectional study performed in communities in Guangzhou, China in 2011 with a participation rate of 98.1% (9916 out of 10,104 eligible individuals signed the consent form). The study was initiated in early 2011, and the collection of baseline screening data was completed by mid-2012. It was based on the Risk Evaluation of cAncers in Chinese diabeTic Individuals: a lONgitudinal (REACTION) study and data have been published previously [13,14,15,16,17]. The study population excluded individuals with type 1 diabetes. A subset of participants (*n* = 317) provided information history of atherosclerotic cardiovascular disease and were excluded from the analysis. This group comprised participants (*n* = 277) with a history of cardiovascular disease, which encompassed cases of myocardial infarction (*n* = 26), and those with a history of stroke (*n* = 53). A total of a total of 555 individuals who failed to provide information (age, *n* = 3; blood pressure (BP), *n* = 81; total cholesterol (TC), *n* = 16; high-density lipoprotein cholesterol (HDL-C), *n* = 20; history of smoking, *n* = 262; history of diabetes, *n* = 3; TGs, *n* = 29; BMI, *n* = 213; γ-GGT, *n* = 58; and WC, *n* = 186) were also excluded as they had missing data points. The details of the selection of study participants are presented in Figure 1.

### 2.2. Patient and Public Involvement

Participants were informed that participation was voluntary and they could withdraw their consent at any time. The results were presented to the participants privately and neither patients nor the public were involved in the design, conducting, reporting, or dissemination of the research. There was no intervention by participants.

### 2.3. Clinical and Biochemical Measurements

In the study, participants’ demographic information and lifestyle factors were obtained through self-reported data. Total physical activity was evaluated by calculating metabolic equivalent hours per week (MET-hour/week) following the International Physical Activity Questionnaire [18].

Anthropometric measurements, including BP, weight, height, and WC, were taken and used to calculate BMI. Diabetes was defined as self-reported use of hypoglycemic medication or a fasting glucose level (FBG) of ≥7.0 mmol/L or 2 h glucose level after oral glucose tolerance test (OGTT) of ≥11.1 mmol/L or HbA1c ≥ 6.5% according to the 2021 American Diabetes Association diagnostic criteria. Hypertension was defined as a systolic BP more than 140 mmHg, and (or) diastolic BP more than 90 mmHg, or self-reported use of oral antihypertensive medication. Drinker referred to individuals who consumed excessive amounts of alcohol, defined as more than 30 g of ethanol per day for men and 20 g of ethanol per day for women according to National Workshop on Fatty Liver and Alcoholic Liver Disease, Chinese Society of Hepatology, Chinese Medical Association [19]. A smoker was defined as an individual who smokes one cigarette a day or seven cigarettes a week for at least six months.

Fasting plasma glucose, insulin, TGs, TC, HDL-C, LDL-C, creatinine (SCr), γ-GGT, aspartate aminotransferase (AST), and alanine aminotransferase (ALT) were measured by autoanalyzer (Beckman CX-7 Biochemical Autoanalyzer, Brea, CA, USA) in fasting samples after at least 10 h overnight, standardized according to IFCC reference procedures [20]. Estimated glomerular filtration rate (eGFR), expressed in mL/min/1.73 m^2^, was calculated using the simplified Modification of Diet in Renal Disease formula recalibrated for the Chinese population. The biochemical analyses were performed using fresh samples on the day the specimens were collected.

FLI was used as a marker of hepatic steatosis, calculated based on BMI, WC, TGs, and γ-GGT, and validated against liver ultrasound in the general population and proven accurate in detecting fatty liver [21,22]. FLI is calculated as follows: FLI  =  (e^0.953 × ln (TG) + 0.139 × BMI + 0.718 × ln (GGT) + 0.053 × WC − 15.745^)/(1  +  e^0.953 × ln (TG) + 0.139 × BMI + 0.718 × ln (GGT) + 0.053 × WC − 15.745^) × 100, with TGs measured in mg/dL (1 mg/dL  =  0.01129 mmol/L), GGT in U/L, and WC in cm. 

### 2.4. Definition of Increased ASCVD Risk and NAFLD

The study used the PCE model [10] to calculate the 10-year ASCVD risk of each participant based on their demographic information and health markers (sex, age, TC, HDL-C, SBP, antihypertensive medication use, smoking status, and presence of diabetes). The 10-year ASCVD risk was classified into low risk (<5%), medium risk (5% to 9%), or high risk (≥10%) [12,23].

The study also used FLI to predict the prevalence of NAFLD. An FLI score of <30 is considered negative for NAFLD, while a score of ≥60 is considered positive in the Western population [22]. The optimal cut-off threshold for FLI to predict NAFLD in the Asian population was found to be 30, with a high sensitivity and specificity of 79.89% and 71.51%, respectively [24]. Participants were classified as having NAFLD if their FLI score was above 30 and as not having NAFLD if their score was below 30.

### 2.5. Statistical Analysis

The statistical analyses were performed using SPSS Version 22 software. The continuous variables were presented as mean ± standard deviation (SD), or as median (inter-quartile range, IQR) while skewed distributed. The categorical variables were expressed as numbers and proportions. The one-way analysis of variance was performed to compare differences among groups, and post-hoc comparisons were conducted using the Bonferroni correction. The Mann–Whitney U test or Kruskal–Wallis test was used to assess the relationships between non-normal variables. To account for the non-normal distribution of certain variables, logarithmic transformation was applied before analysis for ALT, AST, fasting insulin, and physical activity. The χ^2^ test was used to compare categorical variables, the Pearson correlation coefficient was used for continuous variables and treated non-normal variables. Potential covariates that showed significant correlations (*p* < 0.20) in Pearson’s correlations were included in the multivariate stepwise linear regression models to identify factors independently correlated with FLI (while excluding variables that may have exhibited a high correlation with FLI, such as liver enzymes, lipid parameters, and anthropometric parameters).

The independent samples *t* test was applied to compare the differences in continuous variables between two groups. To identify risk factors for ASCVD risk that are independently associated with FLI, multivariate stepwise linear regression models were used. Unadjusted and multivariate-adjusted logistic regression analyses were performed to assess the risk of prevalent ASCVD risk with different FLI levels. Model 1 was unadjusted; Model 2 was adjusted for age; Model 3 was adjusted for age, history of drinking and smoking, and treated hypertension and dyslipidemia; and Model 4 was adjusted for age, SBP, DBP, fasting insulin, HbA1c, SCr, eGFR, HbA1c, history of drinking and smoking, and treated hypertension and dyslipidemia. The results were expressed as odds ratios (ORs) (95% confidence interval, CI), and *p* values less than 0.05 were considered statistically significant.

## 3. Results

### 3.1. General Characteristics of the Participants

The study included 9044 individuals with a mean age of 55.0 ± 7.9 years and 71.6% of whom were female. The median FLI was 18.86 with an IQR of 8.53–37.21. A total of 1298 (14.4%) participants had a 10-year increased ASCVD risk and 458 (5.1%) had a high risk, as assessed by the PCE model on the Assessment of Cardiovascular Risk.

Table 1 illustrates the descriptive characteristics of the participants, divided into different FLI categories. Individuals with higher FLI scores had adverse characteristics including older age; higher levels of metabolic parameters (SBP, diastolic blood pressure (DBP), BMI, WC, TGs, TC, FPG, fasting insulin, and HbA1c); impaired liver function (ALT, AST, and γ-GGT), a higher proportion of current smokers and drinkers; and lower levels of HDL-C, physical activity, and eGFR (all *p* for trend <0.0001).

### 3.2. Association between FLI and Metabolic Risk Factors

Pearson’s correlation analysis showed that 10-year ASCVD risk, age, SBP, DBP, ALT, AST, TC, HDL-C, LDL-C, FPG, fasting insulin, HbA1c, SCr, eGFR, HbA1c, and physical activity were significantly correlated with FLI level (logarithmic transformation was applied before analysis for ALT, AST, fasting insulin, and physical activity). Since FLI is calculated from BMI, TGs, WC, and γ-GGT, we did not investigate these parameters by Pearson’s correlation. Further, excluding variables that may exhibit a high correlation with FLI (such as ALT, AST, TC, HDL-c, or LDL-C), multivariate stepwise linear regression analysis revealed that 10-year ASCVD risk, age, SBP, DBP, FPG, fasting insulin, HbA1c, SCr, eGFR, and HbA1c were independent determinants of FLI level (as shown in Table 2).

### 3.3. Associations of FLI with Increased 10-Year ASCVD Risk

**Figure 2:**
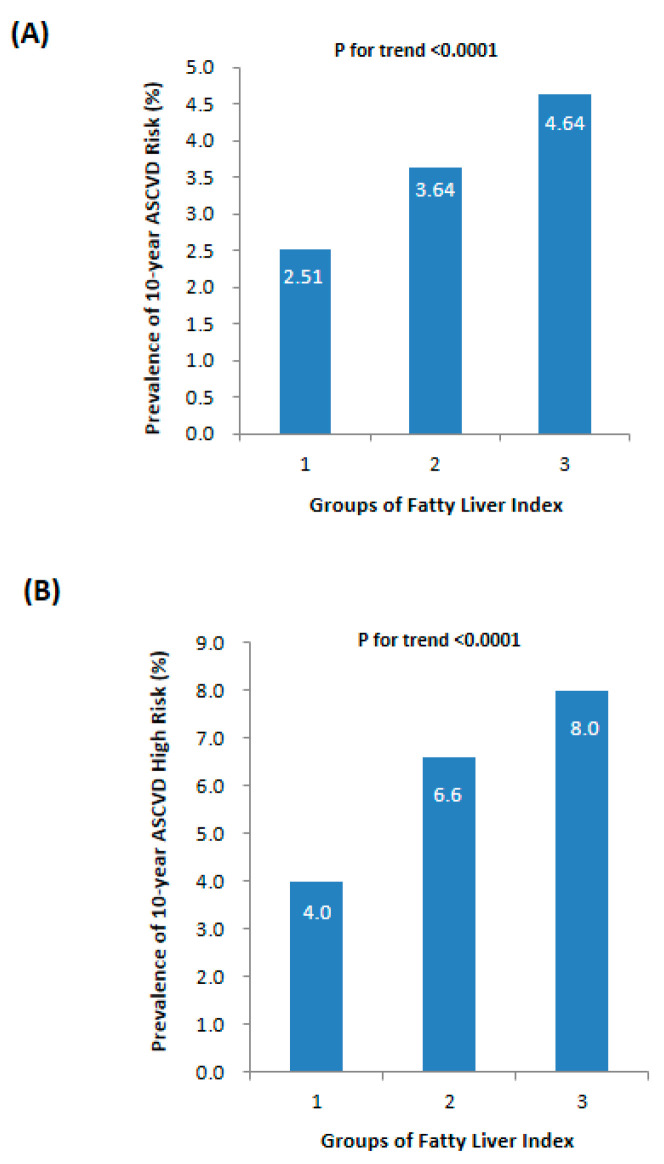
Prevalence of 10-year ASCVD risk in different levels of fatty liver index: (**A**) 10-year ASCVD risk index and (**B**) ratio of 10-year ASCVD high risk.

As illustrated in Figure 2A, there was a progressive increase in the prevalence of 10-year ASCVD risk from the lowest to the highest FLI levels, with rates of 2.51%, 3.64%, and 4.64%, respectively (*p* for trend <0.0001). Similarly, the prevalence of 10-year ASCVD high risk also showed an upward trend with elevated FLI levels (Figure 2B, *p* for trend <0.0001). Table 3 further demonstrated that participants in the FLI < 30 group had a lower prevalence of 10-year ASCVD risk compared to other FLI groups. Univariate logistic regression analysis indicated a significant association between FLI groups and increased odds of 10-year ASCVD risk (all *p* for trend <0.0001). Using the FLI < 30 group as the reference category, participants in the 30 ≤ FLI < 60 group had higher odds of 10-year ASCVD risk with an OR of 1.68 (95% CI 1.36–2.09), while those in the FLI ≥ 60 group had higher odds with an OR of 2.31 (95% CI 1.77–3.00). These correlations persisted even after adjusting for age (Model 2) and non-invasive data of the patients’ medical history, such as a history of drinking and smoking, or treated hypertension and dyslipidemia (Model 3).

In multivariate logistic regression analyses adjusting for age, SBP, DBP, fasting insulin, HbA1c, SCr, eGFR, HbA1c, history of drinking and smoking, and treated hypertension and dyslipidemia (Model 4), the ORs for the prevalence of 10-year ASCVD high risk increased with higher FLI categories, with an OR of 1.00 (reference) for the lowest FLI category, 1.41 (95% CI 0.91–2.18) for the middle FLI category, and 2.61 (95% CI 1.52–4.49) for the highest FLI category. Furthermore, the prevalence of 10-year ASCVD high risk was significantly higher in the FLI-established NAFLD group (8.8%) compared to the non-NAFLD group (4.0%) (*p* < 0.0001). Consistent results were observed in Table 4, which examined the relationship between 10-year ASCVD high risk and FLI levels using different assessment methods (the PCE model, the 2016 China-PAR model [12], and the Framingham Risk Score showed consistent results). When applying The China-PAR model, the ORs for the prevalence of 10-year ASCVD high risk were as follows: FLI < 30 group OR of 1 (reference), 30 ≤ FLI < 60 group OR of 2.77 (95% CI 2.29–3.35), and FLI ≥ 60 group OR of 5.16 (95% CI 4.16–6.38). Similar results were observed when using the classical Framingham Risk Score.

## 4. Discussion

In this study, we analyzed the association between ASCVD and FLI (a marker of NAFLD) in a large number of Chinese individuals from the REACTION study. Our results indicate that higher FLI values were correlated with an increased risk of ASCVD, even after adjusting for multiple potential confounding factors. The intermediate FLI category (30 ≤ FLI < 60) was also independently associated with an increased risk of ASCVD. To the best of our knowledge, this study represents the largest population-based investigation carried out in an Asian population to explore the correlation between FLI and 10-year ASCVD risk. We applied the PCE model of the 2013 ACC/AHA Guideline and the 2016 China-PAR model for the first time to assess this association. The inclusion of the 2016 China-PAR model, which utilizes data from Chinese cohorts, allows for a more precise evaluation of the 10-year ASCVD risk specific to the Chinese population. By integrating these additional assessment models, we have obtained consistent findings that align with the PCE model, Framingham Risk Score, and the China-PAR model. This novel approach provides robust evidence supporting the association between FLI and 10-year ASCVD risk in the Asian population, and underscores the clinical relevance of FLI as a valuable tool for early ASCVD detection in patients with NAFLD.

FLI is a non-invasive indicator representing the accumulation of excess fat in the liver, and has been proven to be an accurate indicator of fatty liver compared to liver ultrasound and magnetic resonance spectroscopy in previous studies [22,25]. It also has been reported in a large-scale population in Asia. According to the study completed by B. Yang et al. [26], the area under the receiver operating characteristic curve (AUROC) for FLI was found to be 0.827 (95% CI, 0.822–0.831). Additionally, another study reported that FLI had high accuracy in identifying NAFLD with an AUROC of 0.834 (95% CI: 0.825–0.842) in China [24]. While there are other biomarkers available for predicting CVD risks in NAFLD patients, such as the comprehensive NAFLD score (CNS) and FLI models for NAFLD [27], they are not widely used in clinical practice due to the inconvenience of measurements and inconsistencies among assays. However, the optimal cut-off point of FLI in assessing NAFLD still needs further exploration, as it may vary in different populations. The commonly used FLI models were primarily formulated in Caucasians, with a cut-off point of >60 [21], but studies have shown that the cut-off value may be lower in Asian populations, such as in a study in Taiwan where the cut-off value was FLI ≥ 35 for males and ≥20 for females [26], and in a study in China where the optimal cut-off point was FLI ≥ 30 in middle-aged and elderly individuals [24]. Further studies are needed to confirm the optimal cut-off value of FLI in the Asian population.

Our study revealed the association between FLI and 10-year ASCVD risk. After adjusting for age, we observed that participants in the 30 ≤ FLI < 60 group had higher odds of 10-year ASCVD risk compared to the FLI < 30 group, with an OR of 1.87 (95% CI 1.32–2.67). Similarly, participants in the FLI ≥ 60 group had even higher odds, with an OR of 3.91 (95% CI 2.52–6.08). These risks continued to exist even after adjusting for metabolic indicators associated with ASCVD. Previous studies have linked FLI to various CVD-related outcomes, including early carotid lesions [28], early coronary lesions [29], arterial stiffness [22], and Framingham risk scores [30]. However, there have been inconsistent results regarding the relationship between FLI and ASCVD. For instance, A. Onat et al. [31] reported a prospective study in Turkish middle-aged adults (*n* = 1822) with a mean 8-year follow-up. They found that FLI ≥ 60 was associated with a higher risk of incident coronary heart disease (CHD) with an adjusted hazard ratio (HR) of 1.72 (95% CI 1.17–2.53). On the other hand, Olubamwo et al. [25] published a prospective study in a European population of 2682 middle-aged and aging men from Eastern Finland, with a median follow-up of 17 years. They reported an association between FLI and incident CVD, but the significance diminished when metabolic factors (SBP, DBP, insulin, FBG, LDL-C, and HDL-C) were included in the analysis, HR was not significant (HR = 1.136, 95% CI: 0.777–1.662). Furthermore, inconsistent results regarding the association between NAFLD and ASCVD have also been reported in different studies. A meta-analysis involving over 34,000 patients [32] demonstrated that individuals with NAFLD have a higher risk of fatal and non-fatal CVD events compared to those without NAFLD (OR: 1.64, 95% CI 1.26–2.13). Another meta-analysis [33] showed that individuals with ultrasound-defined NAFLD had a higher risk of incident cardiovascular disease events (OR: 2.05, 95% CI 1.81 to 2.31) compared to controls without NAFLD. However, there were inconsistent results reported as well. A study of 3529 participants in the US demonstrated that there were no significant correlations between hepatic steatosis and clinical CVD (OR 1.14, 95% CI 0.99–1.32, *p* = 0.07) [34]. A European cohort study of 17.7 million patients reported that NAFLD was not associated with an increased risk of acute myocardial infarction or stroke after adjusting for cardiovascular risk factors [6]. These differences may be due to variations in age, race, sample size, and follow-up time.

These experiments have primarily been conducted in European and American populations. However, there are limited data available on the Asian population. One cross-sectional study conducted in Korea included 7240 individuals aged 30 to 69 years who underwent a health examination between 2015 and 2017 [30]. The study compared individuals with hepatic steatosis (FLI ≥ 60) to those without (FLI < 30) and found that the OR (95% CI) for a high Framingham 10-year cardiovascular disease (CVD) risk ≥ 10% in individuals with hepatic steatosis was 3.43 (3.01–3.91) before adjustment and 2.56 (1.97–3.33) after adjusting for various factors such as age, gender, blood glucose, cholesterol levels, blood pressure, C-reactive protein, lifestyle factors, and smoking status. Another study [29] performed in China investigated the association between FLI and coronary artery disease (CAD). The study included patients with CAD (*n* = 231) and without CAD (*n* = 482) confirmed by coronary angiography. No significant association was found between FLI and CAD in either men (OR: 0.990, 95% CI: 0.980–1.001) or women (OR: 0.988, 95% CI: 0.965–1.012). However, it should be noted that this finding might be underestimated as some patients in the non-CAD group may have had other underlying ASCVD. In our study, which is the largest population-based investigation carried out in an Asian population, we utilized the PCE model, the 2016 China-PAR model, and the Framingham model to assess this association. In a recent cohort study [35] published in JACC: Asia, we included 226,406 participants aged 40 to 79 years without prior ASCVD at baseline from 2010 to 2016 in China. The PCE and China-PAR model demonstrated good discrimination in 5-year ASCVD risk prediction, with the China-PAR model outperforming the PCE model in calibration, making it a better tool for assessing 10-year ASCVD risk in the Chinese population. Consistently, across all three risk prediction models (the PCE model, the China-PAR model, and the Framingham Risk Score), we observed a significant association between FLI and ASCVD, with individuals in the FLI ≥ 60 group showing a significantly higher ASCVD risk compared to those in the FLI < 30 group, providing robust evidence supporting the association between FLI and 10-year ASCVD risk in the Asian population. Overall, these findings suggest that FLI may be an effective screening indicator for the early prevention of ASCVD and related diseases. Further research is needed to confirm the association between NAFLD and CVD in the Asian population and worldwide.

The association between NAFLD and CVD is not surprising, given the close association between NAFLD and established CVD risk factors, such as metabolic factors. Patients with NAFLD often have hypertension and elevated glucose and lipid levels, and are usually overweight or obese. The increase in hepatic lipid accumulation is directly correlated with the severity of each metabolic dysfunction [36]. Our findings, with FLI predicting ASCVD independent of constitutional and metabolic factors, suggest that fatty liver itself may also affect the pathogenesis of CVD through other pathways. Fatty liver ultimately leads to cardiometabolic abnormality and CVD outcomes through a series of pathophysiological processes, including increased oxidative stress, deranged adipokines, deteriorated subclinical inflammation, hypercoagulability, endothelial dysfunction, and atherosclerosis progression [37]. Clinical studies demonstrated that the increasing severity of histologically diagnosed hepatic steatosis is associated with markers of CVD, such as impaired flow-mediated vasodilation, increased carotid intima-media thickness, increased arterial stiffness, and coronary artery calcification [38].

Our study showed that NAFLD is a significant independent factor that contributes to the development of future ASCVD. For early detection, monitoring, and prediction of NAFLD, it is crucial to identify individuals with this condition. In this regard, FLI proves to be a valuable tool, as it is non-invasive, reliable, economical, and easy to calculate. Our findings suggest that FLI has a prognostic value for assessing the risk of ASCVD. It is recommended that individuals with elevated FLI values should undergo an assessment of their ASCVD risk and receive appropriate treatment to prevent cardiovascular disease. Clinicians should pay attention to the identification and management of cardiovascular risk in these individuals during routine care, just as they would for patients with diabetes or hypertension.

This study possesses several strengths and limitations. Remarkably, it is one of the largest population-based studies exploring the correlation between FLI and ASCVD in the Chinese population. A significant strength lies in the utilization of PCE model, the China-PAR model, and the Framingham Risk Score to calculate 10-year ASCVD risks, as these guidelines are widely recognized for their superiority in primary ASCVD prevention and accurate statin-therapy allocation. Moreover, we also applied the Framingham Risk Score and China-PAR model, which is noteworthy as it represents the first use of this model and yielded consistent findings. However, there were still some limitations. Firstly, FLI was used as a surrogate marker of hepatic steatosis [22,25] but was not validated by liver histology or imaging examinations such as CT or MRI. Secondly, this study was cross-sectional and observational, so causal interpretations should be made with caution. Further validation is needed through large prospective studies. The participants were all Chinese, so the results might not be representative of the general population. Thirdly, FLI is a well-validated steatosis score, but it cannot directly evaluate steatohepatitis or liver fibrosis. Further studies should determine the cut-offs of FLI in these conditions. Last but not least, although dietary information was collected, much of it was missing and therefore not reported. Future studies should aim to collect and report comprehensive dietary data to provide a more complete analysis. In light of these limitations, in the future, longitudinal studies are needed to establish causal relationships between FLI, hepatic steatosis, and ASCVD. Validating FLI with liver histology or imaging techniques would further enhance its accuracy as a marker of hepatic steatosis. Additionally, expanding the study population to include diverse ethnic groups and regions would help determine the generalizability of the findings. Furthermore, investigating the relationship between FLI and other liver-related conditions, such as steatohepatitis and liver fibrosis, would provide a more comprehensive understanding of FLI’s clinical utility.

## 5. Conclusions

Our large population-based observational study in China exhibits compelling evidence of the independent correlation between FLI and the prevalence of 10-year ASCVD risk as measured by the PCE model, 2016 China-PAR model, and the Framingham Risk Score. Our findings consistently demonstrate that higher FLI levels are associated with an increased risk of ASCVD. FLI proved to be a valuable tool as a simple, non-invasive, and cost-effective marker of fatty liver and may serve as a useful indicator of cardiovascular diseases. Hence, it is important to pay close attention to cardiovascular diseases in patients with fatty liver disease. Individuals with elevated FLI levels should undergo thorough evaluation and regular monitoring for ASCVD to improve their prognoses. Further research is warranted to explore the potential clinical implications and interventions targeting this high-risk population.

## Figures and Tables

**Table 1:** Characteristics of the study population by FLI.

Characteristics	Total	FLI < 30	30 ≤ FLI < 60	FLI ≥ 60	*p*
*n* (%)	9044 (100.0)	6052 (66.9)	2090 (23.1)	902 (10.0)	
Fatty liver index	18.86 (8.53–37.21)	11.54 (6.03–19.03)	41.37 (34.96–49.48)	71.74 (65.26–80.44)	<0.0001
ASCVD risk (%)	2.99 ± 4.52	2.51 ± 4.11	3.64 ± 4.65 *	4.64 ± 6.04 *^†^	<0.0001
ASCVD HIGH risk, *n* (%)	458 (5.1)	242 (4.0)	137 (6.6) *	79 (8.8) *^†^	<0.0001
Age (yr)	55.0 ± 7.9	54.6 ± 7.8	56.0 ± 8.0 *	55.8 ± 8.2 *	<0.0001
Male, *n* (%)	2572 (28.4)	1396 (23.1)	751 (35.9) *	425 (47.1) *^†^	<0.0001
BMI (kg/m^2^)	23.60 ± 3.15	22.30 ± 2.45	25.50 ± 2.27 *	27.88 ± 3.07 *^†^	<0.0001
WC (cm)	81.6 ± 9.5	77.5 ± 7.2	87.6 ± 6.0 *	95.2 ± 9.4 *^†^	<0.0001
SBP (mm Hg)	125.9 ± 16.5	123.0 ± 15.8	130.9 ± 15.7 *	134.4 ± 17.1 *^†^	<0.0001
DBP (mm Hg)	75.3 ± 9.9	73.5 ± 9.4	78.3 ± 9.7 *	80.7 ± 10.0 *^†^	<0.0001
Treated hypertension, *n* (%)	808 (8.9)	405 (6.7)	252 (12.1) *	151 (16.7) *^†^	<0.0001
ALT (U/L)	13.0 (9.0–18.0)	12.0 (9.0–16.0)	15.0 (11.0–20.0) *	19.0 (13.0–28.0) *^†^	<0.0001
AST (U/L)	18.0(15.0–22.0)	18.0 (14.0–21.0)	19.0 (16.0–23.0) *	22.0 (18.0–27.0) *^†^	<0.0001
γ-GGT (U/L)	20.0 (14.0–28.0)	17.0 (13.0–22.0)	26.0 (20.0–36.0) *	40.0 (27.0–63.0) *^†^	<0.0001
TGs (mmol/L)	1.59 ± 1.25	1.18 ± 0.53	2.05 ± 1.09 *	3.34 ± 2.53 *^†^	<0.0001
TC (mmol/L)	5.22 ± 1.22	5.07 ± 1.23	5.44 ± 1.15 *	5.67 ± 1.23 *^†^	<0.0001
HDL-C (mmol/L)	1.32 ± 0.36	1.39 ± 0.37	1.23 ± 0.29 *	1.16 ± 0.35 *^†^	<0.0001
LDL-C (mmol/L)	3.16 ± 0.95	3.09 ± 0.94	3.31 ± 0.95 *	3.28 ± 0.99 *	<0.0001
Treated dyslipidemia, *n* (%)	96 (1.1)	57 (0.9)	26 (1.2)	13 (1.4)	0.256
FPG (mmol/L)	5.68 ± 1.34	5.49 ± 1.14	5.91 ± 1.40 *	6.38 ± 2.00 *^†^	<0.0001
Fasting Insulin (μIU/mL)	7.10 (5.20–9.90)	6.20 (4.60–8.20)	9.10 (7.00–12.10) *	11.70 (8.90–15.40) *^†^	<0.0001
HbA1c (%)	6.04 ± 0.90	5.92 ± 0.78	6.22 ± 0.99 *	6.48 ± 1.22 *^†^	<0.0001
Diabetes, *n* (%)	623 (6.9)	352 (5.8)	174 (8.3) *	97 (10.8) *^†^	<0.0001
SCr (μmol/L)	69.8 ± 16.7	67.9 ± 16.3	72.4 ± 15.1 *	76.9 ± 20.0 *^†^	<0.0001
eGFR (ml/min per 1.73 m^2^)	112.9 ± 23.3	115.0 ± 24.3	109.6 ± 19.9 *	106.6 ± 22.0 *^†^	<0.0001
Physical activity (MET-hour/week)	21.0 (10.5–45.0)	24.0 (10.5–46.0)	21.0 (10.5–42.0) *	21.0 (9.0–45.1) *	0.013
Current smoker, *n* (%)	924 (10.2)	518 (8.6)	245 (11.7) *	161 (17.8) *^†^	0.032
Drinker, *n* (%)	65 (0.7)	26 (0.4)	21 (1.0) *	18 (2.0) *^†^	<0.001

Data are mean ± SD or median (IQR) for skewed variables or numbers (proportions) for categorical variables. *p* values are for the analysis of variance or χ^2^ analyses across the groups. The Mann–Whitney U test or Kruskal–Wallis test was used to assess the relationships between non-normal variables. * *p* < 0.05 compared with the group of fatty liver index < 30. ^†^ *p* < 0.05 compared with the group of 30 ≤ fatty liver index < 60. ALT: alanine aminotransferase; ASCVD: atherosclerotic cardiovascular disease; ASCVD HIGH risk: 10-year ASCVD risk prediction ≥10%; ASCVD risk: 10-year ASCVD risk prediction; AST: aspartate aminotransferase; BMI: body mass index; DBP: diastolic blood pressure; drinker: individual who consumes excessive amounts of alcohol; eGFR: estimated glomerular filtration rate; FPG: fasting plasma glucose; FLI: fatty liver index; γ-GGT: gamma-glutamyl transpeptidase; HDL-C: high-density lipoprotein cholesterol; LDL-C: low-density lipoprotein cholesterol; MET-hour/week: metabolic equivalent hours per week; SBP: systolic blood pressure; SCr: serum creatinine; TC: total cholesterol; TG: triglycerides; WC: waist circumference.

**Table 2:** Correlation analysis among characteristics of the study population and FLI.

	r	*p*	Standardized β	*p*
ASCVD risk (%)	0.183	<0.0001	0.076	0.004
Age (yr)	0.092	<0.0001	−0.039	0.004
BMI (kg/m^2^)	0.716	<0.0001		
WC (cm)	0.758	<0.0001		
SBP (mm Hg)	0.299	<0.0001	0.036	0.014
DBP (mm Hg)	0.306	<0.0001	0.146	<0.0001
ALT (U/L)	0.348	<0.0001		
AST (U/L)	0.246	<0.0001		
GGT (U/L)	0.405	<0.0001		
TC (mmol/L)	0.216	<0.0001		
TGs (mmol/L)	0.602	<0.0001		
HDL-C (mmol/L)	−0.264	<0.0001		
LDL-C (mmol/L)	0.139	<0.0001		
FPG (mmol/L)	0.241	<0.0001	0.030	0.032
Fasting insulin (μIU/mL)	0.570	<0.0001	0.445	<0.0001
SCr (μmol/L)	0.208	<0.0001	0.186	<0.0001
eGFR (ml/min per 1.73 m^2^)	−0.160	<0.0001	0.040	0.003
HbA1c (%)	0.238	<0.0001	0.098	<0.0001
Physical activity (MET-hour/week)	−0.027	0.012	−0.008	0.404

For variables that follow a normal distribution, Pearson’s correlation and stepwise regression analysis were conducted to examine the determinants of FLI. For variables that do not follow a normal distribution, including ALT, AST, fasting insulin, and physical activity, logarithmic transformation was applied before analysis. In the multivariate stepwise linear regression analysis, variables that were found to have a high correlation with FLI (including ALT, AST, TC, HDL-C, and LDL-C) were excluded.

**Table 3:** Odds ratios for prevalent 10-year ASCVD high risk (≥ 10%) according to the fatty liver index.

		FLI < 30	30 ≤ FLI < 60	FLI ≥ 60	*p*
10-year ASCVD HIGH risk	MODEL 1	1	1.68 (1.36–2.09)	2.31 (1.77–3.00)	<0.0001
	MODEL 2	1	1.87 (1.32–2.67)	3.91 (2.52–6.08)	<0.0001
	MODEL 3	1	1.89 (1.28–2.77)	3.88 (2.40–6.28)	<0.0001
	MODEL 4	1	1.41 (0.91–2.18)	2.61 (1.52–4.49)	<0.0001

Data are ORs (95% CI). Participants without 10-year ASCVD HIGH risk are defined as 0 and with 10-year ASCVD HIGH risk as 1. Model 1 is unadjusted. Model 2 is adjusted for age. Model 3 is adjusted for age, a history of drinking and smoking, and treated hypertension and dyslipidemia. Model 4 is adjusted for age, SBP, DBP, fasting insulin, HbA1c, SCr, eGFR, HbA1c, history of drinking and smoking, and treated hypertension and dyslipidemia.

**Table 4:** Odds ratios for prevalent 10-year ASCVD high risk (≥10%) in different models according to fatty liver index.

	FLI < 30	30 ≤ FLI < 60	FLI ≥ 60	*p*
The PCE model	1	1.68 (1.36–2.09)	2.31 (1.77–3.00)	<0.001
The China-PAR model	1	2.77 (2.29–3.35)	5.16 (4.16–6.38)	<0.001
The Framingham Risk Score	1	2.31 (2.02–2.65)	3.66 (3.10–4.32)	<0.001

Data are ORs (95% CI). Participants without 10-year ASCVD HIGH risk are defined as 0 and with 10-year ASCVD HIGH risk as 1.

## Data Availability

Main document data and additional unpublished data from the study are available by sending an email to zhoujing23@mail.sysu.edu.cn outlining the purpose of the request due to privacy or ethical restrictions.

## Abbreviations

ACC/AHA: American College of Cardiology/American Heart Association; ALT: alanine aminotransferase; ASCVD: atherosclerotic cardiovascular disease; ASCVD HIGH risk: 10-year ASCVD risk prediction ≥10%; ASCVD risk: 10-year ASCVD risk prediction; AST: aspartate aminotransferase; BMI: body mass index; DBP: diastolic blood pressure; eGFR: estimated glomerular filtration rate; FLI: fatty liver index; FPG: fasting plasma glucose; γ-GGT: gamma-glutamyl transpeptidase; HDL-C: high-density lipoprotein cholesterol; LDL-C: low-density lipoprotein cholesterol; MET-hour/week: metabolic equivalent hours per week; NAFLD: Non-alcoholic fatty liver disease; NASH: non-alcoholic steatohepatitis; SBP: systolic blood pressure; SCr: serum creatinine; TC: total cholesterol; TGs: triglycerides; US-PCE: United States Pooled Cohort Equations; WC: waist circumference.
